# Comparative Evaluation of Agroindustrial Byproducts for the Production of Alkaline Protease by Wild and Mutant Strains of *Bacillus subtilis* in Submerged and Solid State Fermentation

**DOI:** 10.1155/2013/538067

**Published:** 2013-10-31

**Authors:** Hamid Mukhtar, Ikramul Haq

**Affiliations:** Institute of Industrial Biotechnology, GC University, Lahore 54000, Pakistan

## Abstract

The present study describes the screening of different agroindustrial byproducts for enhanced production of alkaline protease by a wild and EMS induced mutant strain of *Bacillus subtilis* IH-72^EMS8^. During submerged fermentation, different agro-industrial byproducts were tested which include defatted seed meals of rape, guar, sunflower, gluten, cotton, soybean, and gram. In addition to these meals, rice bran, wheat bran, and wheat flour were also evaluated for protease production. Of all the byproducts tested, soybean meal at a concentration of 20 g/L gave maximum production of the enzyme, that is, 5.74  ±  0.26 U/mL from wild and 11.28  ±  0.45 U/mL from mutant strain, during submerged fermentation. Different mesh sizes (coarse, medium, and fine) of the soybean meal were also evaluated, and a finely ground soybean meal (fine mesh) was found to be the best. In addition to the defatted seed meals, their alkali extracts were also tested for the production of alkaline protease by *Bacillus subtilis*, but these were proved nonsignificant for enhanced production of the enzyme. The production of the enzyme was also studied in solid state fermentation, and different agro-industrial byproducts were also evaluated for enzyme production. Wheat bran partially replaced with guar meal was found as the best substrate for maximum enzyme production under solid state fermentation conditions.

## 1. Introduction

Microorganisms represent an excellent source of proteolytic enzymes owing to their broad biochemical diversity and their suitability to genetic manipulation. Microbial proteases account for approximately 40% of the total worldwide enzyme sales [1]. Proteases from microbial sources are preferred to the enzymes from plant and animal sources, since they possess almost all the characteristics desired for their biotechnological applications. In addition, the microbial enzymes are not subjected to any of the production and supply limitations. 

Alkaline proteases are produced by a large number of bacterial species; however, *Bacillus* species possess remarkable biotechnological value due to their nonpathogenicity of various species and the ability to produce extracellular protease in large amounts. Different species of *Bacillus* producing high titers of protease include *B. subtilis* and *B. licheniformis* [2, 3], *B. pseudofirmus* [4], *B. cereus*, *B. pumilus* [5], *B. stearothermophilus* [6], *B. intermedius* [7], *B. amyloliquefaciens* [8], and *Bacillus mycoides* [9]. Protease production has also been reported by alkalophilic *Streptomyces* spp. [10], *Pseudomonas* spp. [11], *Photorhabdus* spp. [12], *Lactobacillus* spp. [13], *Alcaligenes faecalis* [14], and *Xanthomonas maltophilia* [15].

Proteases can be obtained economically and profitably from microorganisms by the process of fermentation using submerged as well as solid state fermentation. Many of the organisms excrete more than one kind of protease, and the type of proteolytic enzyme formed may depend upon the composition of the medium [16]. The agroindustrial byproducts when used in the submerged culture medium are excellent and cheap sources of proteins, carbohydrates, and minerals needed for the growth of microorganisms and synthesis of microbial enzymes. Solid state fermentation (SSF) has the potential to serve as a production method for microbial products and processes typically using agricultural crop and processing residues. Such substrates are structurally and mutationally complex, often creating a supportive environment compared to the submerged cultivation method. 

Pakistan is an agricultural country so it has wide variety of agroindustrial byproducts which are cheaply available in the market. These agroindustrial by-products, being a good source of proteins, carbohydrates, and minerals, can be exploited for the biosynthesis of industrial enzymes through microorganisms. These substrates include agricultural crop and processing residues such as wheat bran, soybean meal, sugar cane bagasse, corn stover, residues of coffee, paper and oil processing, cereal brans and husks, and different defatted oil seed cakes. Bacteria generally synthesize proteolytic enzymes, when grown in protein medium, however, very few bacteria can also produce proteases on protein-free media [16], and these agroindustrial by-products present a wide variety of protein rich substrates.

 The present study was undertaken to evaluate the indigenously available cheap agroindustrial resources for the production of alkaline protease from a locally isolated mutant strain of *Bacillus subtilis* using both the submerged and solid state fermentations.

## 2. Materials and Methods

### 2.1. Microorganism

The microorganism used in the present study was *Bacillus subtilis* IH-72 which was originally isolated from the soil of tannery area and identified in our labs. The wild strain has been maintained in the culture bank of the Institute of Industrial Biotechnology, GC University, Lahore.

### 2.2. Random Mutagenesis

The  random mutagenesis of the wild strain of *Bacillus subtilis* was carried out using ethyl methane sulfonate (EMS) for enhanced production of proteases according to the protocols developed in our labs. The wild culture  was subcultured in nutrient broth medium overnight until a density of 3–5 × 10^8^ CFU/mL was reached. The culture was then centrifuged at 6000 rpm for 10 min. The pellet was washed with sterilized saline water and resuspended in half the original volume of nutrient broth containing 0.2 M Tris (pH 7.5). EMS was then added to the bacterial suspension to the final concentration of 15 ul/mL and mixed vigorously. Then the tubes were incubated in shaking water bath at 37°C for 30–180 min. After incubation, the culture was centrifuged and washed with sterilized saline water. The pellet was resuspended in 10 mL of nutrient broth and allowed to grow overnight. The saturated culture was diluted up to 10^6^ and plated on peptone-yeast extract-casein agar plates. The plates were incubated at 37°C for 24–48 hrs for growth of the mutagenized cells. The bacterial colonies showing bigger zones of casein hydrolysis as compared to wild strain were picked up and transferred to the nutrient broth slants. All the isolated mutants were screened for enhancement in the enzyme production (data not shown) and the best mutant strain namely *Bacillus subtilis*  IH-72^EMS8^ was selected for further studies.

### 2.3. Fermentation Experiments

The fermentation experiments for the production of extracellular alkaline protease by *Bacillus subtilis* IH-72 were carried out in flasks as solid state and submerged fermentation and laboratory scale stirred fermenter. 

### 2.4. Inoculum Preparation

Fifty milliliter of preculture medium consisting of 0.8% nutrient broth (peptone, 0.3%; yeast extract, 0.4%; pH 7) was transferred to the cotton-plugged 250 mL Erlenmeyer flask and sterilized in an autoclave for 15 min at 15 Lb/inch^2^ (121°C). After cooling at room temperature, the flask was inoculated aseptically with a loopful of bacteria from 48 hrs old slant. The flask was then placed in the rotary shaking incubator at 37°C for 24 hrs. The bacterial growth was used as an inoculum in both the submerged and solid state fermentations.

### 2.5. Submerged Fermentation

Fifty milliliter of the fermentation medium composed of (g/L) soybean meal, 10; glucose, 10; polypeptone, 10; KH_2_PO_4_, 1.0; and Na_2_CO_3_, 5.0 (pH 8.5) contained in 250 mL cotton-plugged Erlenmeyer flask was sterilized in an autoclave for 15 min at 15 lb/inch^2^ (121°C) and cooled at room temperature. Each flask was then inoculated with 1 mL of vegetative inoculum of bacterial cells containing 5.2-5.3 × 10^8^  × CFU/mL. The flasks were then placed in the rotary shaking incubator (200 rpm) at 35°C for 48 hrs. After a fixed incubation period, the fermented broth was centrifuged at 5000 rpm for 10 min. The supernatant was analyzed for protease assay and estimation of dry cell mass.

### 2.6. Solid State Fermentation

For solid state fermentation, 5 g of wheat bran and 5 g of soybean meal contained in 250 mL Erlenmeyer flask were moistened with 10 mL of distilled water. The flasks were cotton-plugged and sterilized in an autoclave. After sterilization, the medium was cooled at room temperature and was inoculated with 1.0 mL of the bacterial inoculum as prepared earlier. The flasks were vigorously shaken to distribute the inoculum uniformly in the medium and were incubated statically at 37°C for 48 hrs. During incubation, the flasks were shaken twice a day for achieving homogeneity. 

The fermetation batches were run in triplicate, and the mean of three was reported in the results. During all the experiments, calibrated glassware and analytical grade chemicals were used.

## 3. Analytical Methods 

### 3.1. Assay of Protease

The method of McDonald and Chen [17] was used for the assay of protease. Casein (1% solution in 0.1 M phosphate buffer of pH 8.0) was incubated with the enzyme sample at 30°C for 30 min. The reaction was arrested by the addition of 5 mL of 5% trichloroacetic acid (TCA) solution. The mixture was centrifuged at 5000 rpm for 10 min, and 1 mL of supernatant was mixed with 5 mL of alkaline regent. To this mixture 1 mL of 1 N NaOH was added to make the contents of the tube alkaline. After 10 min., 0.5 mL of Folin and Ciocalteau reagent was added to the test tubes and mixed. The blue colour produced was measured with UV-VIS spectrophotometer at 700 nm after 30 min.

One unit of protease activity is defined as the amount of enzyme required to produce and increase 0.1 in optical density at 700 nm under the defined conditions.

Abbreviations used: RSM: rape seed meal; Gl.M: gluten meal; GM: guar meal; Sf.M: sunflower meal; CSM: cotton seed meal; SBM: soybean meal; WB: wheat bran; WF: wheat flour; FM: fish meal.

## 4. Results 

### 4.1. Screening of Agroindustrial Substrates during Submerged Fermentation

**Figure 1:**
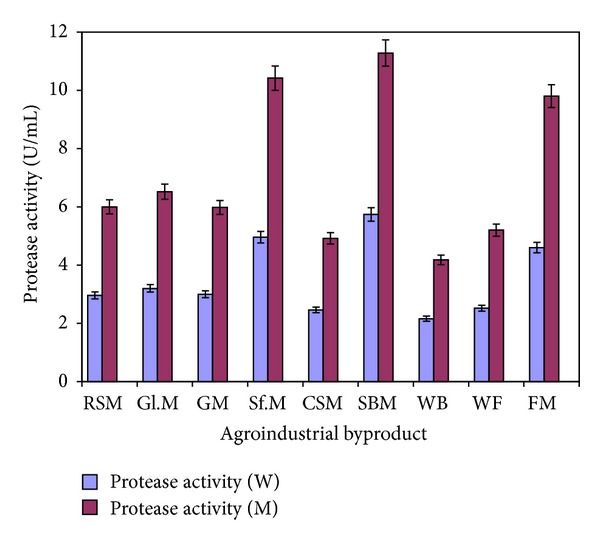
Screening of different agroindustrial byproducts for the production of alkaline protease by *Bacillus subtilis* IH-72 and its mutant derivative in shake flasks. (Initial pH 8.5; incubation temperature 37°C; fermentation period (W = wild) 48 hrs; fermentation period (M = mutant) 42 hrs; fermentation medium M4. Each value is an average of three parallel replicates. Y bars indicate the standard error of mean value.) RSM: rape seed meal; Gl.M: gluten meal; GM: guar meal; Sf.M: sunflower meal; CSM: cotton seed meal; SBM: soybean meal; WB: wheat bran; WF: wheat flour; FM: fish meal.

Figure 1 depicts the screening of different agroindustrial byproducts for the production of alkaline protease by wild and mutant strains of *Bacillus subtilis* in submerged fermentation. Of all the agroindustrial byproducts evaluated, soybean meal was found to be the best substrate for production of alkaline protease showing a yield of 5.74 ± 0.26 U/mL (wild) and 11.28 ± 0.45 U/mL (mutant). The other substrates such as rape seed meal, gluten meal, guar meal, sunflower meal, cotton seed meal, wheat bran, wheat flour, and fish meal gave 2.96 ± 0.12, 3.2 ± 0.13, 3.0 ± 0.12, 4.9 ± 0.196, 6.0 ± 0.24, 2.46 ± 0.098, 2.16 ± 0.86, and 2.52 ± 0.10 U/mL with wild strain and 6.0 ± 0.24, 6.52 ± 0.26, 5.9 ± 0.24, 8.0 ± 0.32, 10.42 ± 0.42, 4.92 ± 0.196, 4.18 ± 0.167, and 5.2 ± 0.21 U/mL of alkaline protease with mutant strain, respectively. The alkali extracts of abovementioned seed meals were also evaluated for the production of alkaline protease by *Bacillus subtilis*, but these were proved nonsignificant for enhanced production of the enzyme (data not shown).

**Figure 2:**
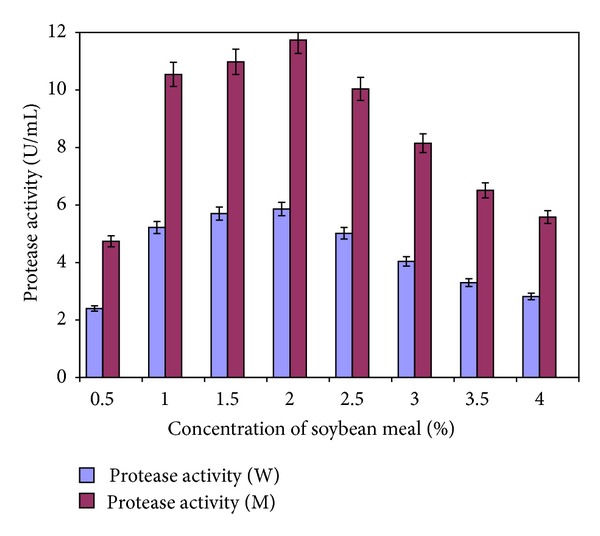
Effect of different concentrations of soybean meal on the production of alkaline protease by *Bacillus subtilis* IH-72 and its mutant derivative in shake flasks. (Initial pH 8.5; incubation temperature 37°C; fermentation period (W) 48 hrs; fermentation period (M) 42 hrs; fermentation medium M4. Each value is an average of three parallel replicates. Y bars indicate the standard error of mean value.)

In continuation, further experiments were performed to find out the optimum concentration of soybean meal in the fermentation medium for the production of alkaline protease by the wild and mutant organism (Figure 2). The soybean meal was added to the culture medium at different concentrations ranging from 0.5 to 4.0% (w/v). The results showed that the soybean meal at a concentration of 2% was best for maximum biosynthesis of alkaline protease (5.86 ± 0.23 U/mi (W) and 11.74 ± 0.47 U/mL (M)). Lesser or greater concentration of soybean meal than 2% (w/v) resulted in a decreased amount of alkaline protease production by the microorganism.

**Figure 3:**
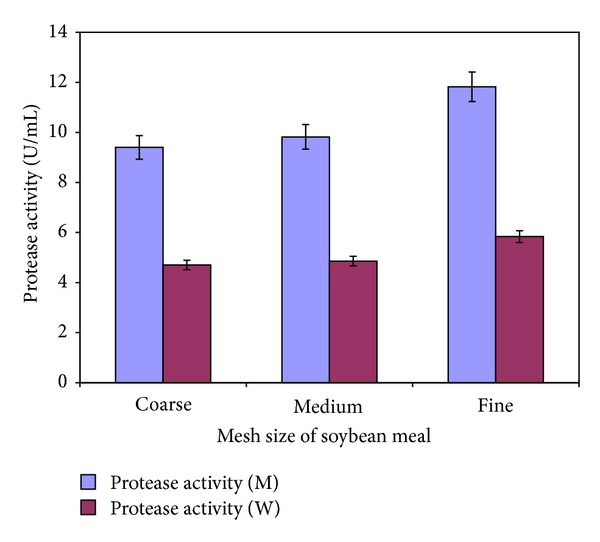
Effect of mesh size of soybean meal on the production of alkaline protease by *Bacillus subtilis* IH-72 and its mutant derivative in shake flasks. (Initial pH 8.5; incubation temperature 37°C; fermentation period (W) 48 hrs; fermentation period (M) 42 hrs; fermentation medium M4. Each value is an average of three parallel replicates. Y bars indicate the standard error of mean value.)

In another set of experiments, the mesh size of soybean meal was optimized for maximum biosynthesis of alkaline protease by both the wild and mutant strains of *Bacillus subtilis* IH-72 (Figure 3). Soybean meal in three different mesh sizes (60, 100, and 150) was added to the culture medium and fermentation was carried out. The results of the experiments as shown in Figure 3 depicted that the finely ground soybean meal (60 mesh) supported maximum growth of the microorganism and production of enzyme in case of both the wild (5.84 ± 0.25 U/mL) and mutant (11.82 ± 0.48) so was best for biosynthesis of the enzyme. Therefore, the culture medium was supplemented with soybean meal having a mesh size of 60 at a concentration of 2% for maximum production of alkaline protease by *Bacillus subtilis* IH-72 and its mutant derivative.

### 4.2. Screening of Agroindustrial Substrates during Solid State Fermentation

**Figure 4:**
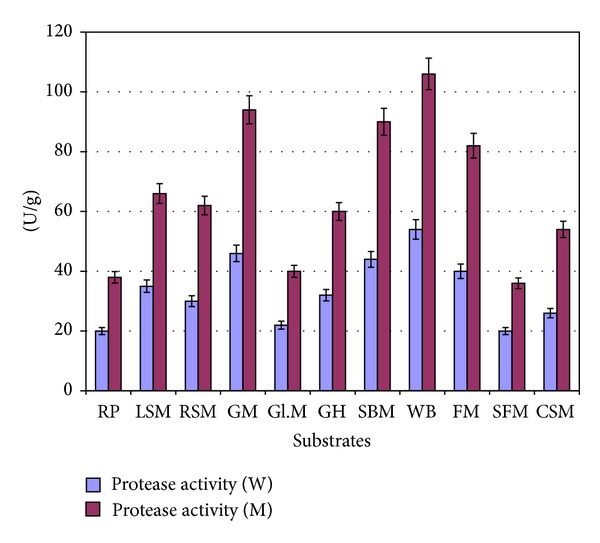
Screening of different agroindustrial byproducts for the production of alkaline protease by *Bacillus subtilis* IH-72 and its mutant derivative using solid state fermentation. (Incubation temperature = 37°C; incubation period = 48 hrs; moisture level = 100%; diluent =  distilled water; pH of diluent 7.0; inoculum size 10%.) Each value is a mean of three replicates. Y error bars indicate the standard error from the mean. RP: rice polish; LSM: linseed meal; RSM: rape seed meal; GM: guar meal; Gl.M: gluten meal; GH: gram husk; SBM: soybean meal; WB: wheat bran; FM: fish meal; SFM: sunflower meal; CSM: cotton seed meal.

Selection of a suitable substrate for solid state fermentation is of great importance for a successful fermentation process. Different substrates such as rice polish, linseed meal, rape seed meal, guar meal, cotton gluten meal, sunflower meal, gram husk, soybean meal, wheat bran, and fish meal were evaluated as source of protein, carbohydrate, and minerals for the synthesis of protease by *Bacillus subtilis* IH-72 under solid state fermentation conditions.  Figure 4 shows that out of all the fermentation substrate examined, wheat bran gave maximum enzyme production, that is, 85.03 U/g. It was decreased in the order guar meal (79.25 U/g) > soybean meal (53.15 U/g) > gram husk (40.69 U/g) > linseed meal (35.28 U/g) > sunflower meal (32.45 U/g) > rape seed meal (28.20 U/g) > cotton gluten meal (22.1 U/g) > rice polish (20.50 U/g) > fish meal (20.0 U/g).

**Figure 5:**
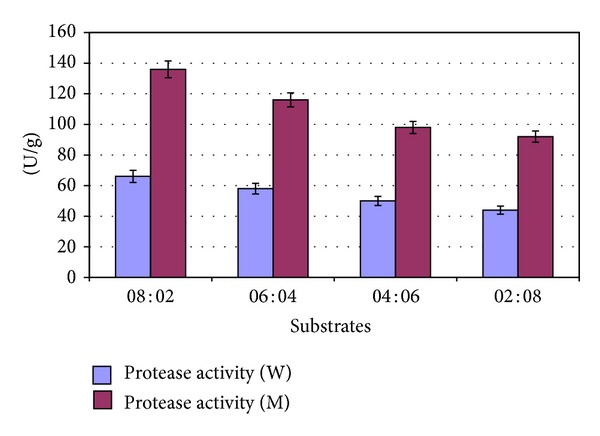
Effect of partial replacement of wheat bran with guar meal on the production of alkaline protease by *Bacillus subtilis* IH-72 using solid state fermentation. (Incubation temperature = 37°C; incubation period = 48 hrs; moisture level = 100%; diluent = distilled water; pH of diluent = 7.0; inoculum size = 10%.) Each value is a mean of three replicates. Y error bars indicate the standard error from the mean.

As the biosynthesis of alkaline protease was found maximum in the presence of wheat bran and guar meal was next to wheat bran in this regard (Figure 4), so wheat bran was partially replaced with guar meal for maximizing the enzyme production. It was found that the alkaline protease formation was maximum (89.15 U/g) when wheat bran was replaced with guar meal at a ratio of 8 : 2 (Figure 5). Further replacement of wheat bran with guar meal resulted in the decreased production of protease because it reduced the porosity of substrate and resulted in the scum formation.

## 5. Discussion

The selection of an ideal agroindustrial byproduct for enzyme production depends on several factors, which are mainly related to cost and availability of the material and thus may involve screening of several such byproducts. During submerged fermentation, different agroindustrial byproducts were evaluated for the production of alkaline protease by the organism, and soybean meal, which is a byproduct of oil mills, was found to be the best protein substrate for the induction of protease production by *Bacillus subtilis* IH-72. Soybean meal contains (% s/s) protein, 45; carbohydrates, 32.2; fat, 0.8; Ca, 0.25; Mg, 0.27; P, 0.6; K, 1.92; and S, 0.32, in addition to the vitamins and amino acids (Traders Protein, USA). It is evident that soybean meal contains good amount of protein and other nutrients which are supportive of profuse growth and secretion of proteolytic enzyme. Several other workers have also used soybean meal as a substrate in the medium for induction of proteases by *Bacillus* species [18, 19]. On the other hand, some proteo-chitinous (milled shrimp waste) and nonproteinaceous substrates have also been used to produce protease by *Bacillus* spp. [16, 20].

The reason for the highest yield with wheat bran in case of solid state fermentation was due to the fact that it provided an adequate source of protein, carbohydrates, and minerals needed by the microorganism for growth and biosynthesis of protease. It also had a large surface area per unit volume for a good bacterial growth on the solid/gas interfaces. The superior effect of natural substrates in enzyme production may also be due to the presence of growth promoters in enough amounts covering the requirements of the bacterial growth and enzyme production. Several other workers have also reported wheat bran as the best substrate to yield a higher enzyme production from *Bacillus* species under solid state fermentation [21, 22]. *Bacillus horikoshii*, *B. subtilis*, and *B. circulans* have been reported to synthesize the maximum alkaline proteases using soybean meal, rice bran, and green gram husk, respectively [18, 23].

The increased production of protease with wheat bran in combination with guar meal may be due to the fact that wheat bran alone may be deficient in some nutrients and guar meal might have fulfilled the deficient nutritional requirement of the organism for growth. Resulted fermentation substrate became very supportive for microbial growth and yield of alkaline protease by *Bacillus subtilis* IH-72. It was also reported that *Bacillus subtilis* gave maximum yield of alkaline protease when wheat bran was partially replaced with soybean meal (9 : 1) as compared with other substrates [24].

## 6. Conclusion

The present study concludes that the cheaply available industrial byproducts have a good potential and can be used as substrates for the production of different value-added products such as commercial enzymes from different microorganisms using either submerged or solid state fermentations.
